# A Tale of Two Principles—Towards a Relational Perspective on the Free Energy Principle

**DOI:** 10.3390/e28101103

**Published:** 2026-10-10

**Authors:** Avel Guénin--Carlut

**Affiliations:** 1 School of Engineering and Informatics, University of Sussex, Brighton BN1 9RH, UK; avel@kairos-research.org; 2 Active Inference Institute, Crescent City, CA 95531, USA; 3 Kairos Research, 18190 Châteauneuf-sur-Cher, France

**Keywords:** Free Energy Principle, active inference, Markov blanket, contextuality, participatory realism, biological autonomy, relational biology, structural realism, thermodynamics of computation, constraints closure

## Abstract

The Free Energy Principle (FEP) offers a physically grounded account of intentional properties in mind and life, framed in the context of Dynamical Systems Theory. Because of this generality, it was proposed as a formal basis to formalize the study of biological autonomy, autopoiesis, and enactive cognition. Yet, those approaches are centered by definition on describing how biological and cognitive agents are “laying down a path in walking”, or (re)constructing their own biological/cognitive structure through engagement with the world. Drawing from Rosen’s relational biology, we argue that such processes are by construction outside a “Newtonian” description in terms of well-defined observables and their dynamics. Rather, we draw from the thermodynamics of observation and biological computation to demonstrate the emergence of *contextuality*: the participatory production of observables against which the agent regulates their activity. We suggest a complementarity between Friston’s classical FEP and Fields’ relational FEP, describing, respectively, the dynamics within a biological/cognitive space and the construction of that space. Finally, we propose a structural realist understanding of this complementarity where both principles act on a physically embedded system of biological constraints, rather than an abstract mathematical space assumed to fully represent biological/cognitive organization.

## 1. Introduction

The recent years have seen the emergence of a fierce debate regarding the adequacy of Friston’s [1,2,3,4,5,6] Free Energy Principle (FEP) to represent life and cognition. In its modern form [6], the FEP indeed suggests a quite seductive proposal: mathematically grounding a Bayesian model of perception and action into the bare dynamics of the agent–world system, under rather minimal conditions on the structural and statistical properties of the interface. This mathematical framework was understood by many to dissolve important open problems in cognitive science, going from computational limits on perceptual inference and action planning [7,8] to the conflict between “representational” and “enactive” or “embodied” approaches to cognition [9,10,11,12,13,14] or the articulation between mind and culture [15,16,17,18,19]. More provocatively, some presented the FEP as a principled theory of every single “thing”, in the sense that, to constitute a “thing”, any natural system should follow its axioms [6,20,21,22]. Unsurprisingly, many researchers found it relevant to comment on the epistemological basis of these claims, taking every conceivable position from direct realism to variants of instrumentalism about the FEP [23,24,25,26,27,28,29].

Our present discussion is concerned with a subregion of this general domain of enquiry, if an especially active one. Indeed, Friston [3,5] proposed the FEP as a general theory of life, a claim that was repeated (and reinforced) by multiple proponents [20,22,30]. In particular, Kirchhoff and Froese [31], Allen and Friston [32], Kirchhoff [33], Kirchhoff et al. [34], Wiese and Friston [35], and Guénin--Carlut [36] suggested that the FEP reproduced the core insight of Varela’s [37] theory of biological organization: autopoiesis. This suggestion had quite important stakes for the legitimacy of the FEP as a whole, as autopoiesis grounded Varela et al.’s [38] very own enactive approach to cognition—as we discussed above, an important target to meet for proponents of the FEP. This claim, however, was not well received by most contemporary proponents of the enactive approach. Di Paolo et al. [39], while acknowledging the relevance of the FEP in its own right, outline a “forking path” between the two approaches given their different perspectives on historicity, agency, and most importantly the self-constitutive aspect of life. Jaeger et al. [40] denied that the enactive conception of meaning was compatible with a computational approach to life and mind. Nave [41,42] argued that the FEP fails to properly account for the thermodynamic processes driving the construction of biological symmetries, thus failing to properly ground the emergence of biological organization. These claims can appear astounding to the contemporary naturalist: what would living systems be made of, what would cognition emerge from, if not physical particles following a specific law of motion?

The answer to this question is to be found in Varela’s [37] very own articulation of autopoietic theory. To Varela [37], there is an important conceptual distinction to be made between the *structure* of a system (“the actual relations that hold between the components that integrate a concrete [system] in a given space”, p. 16) and its *organization* (“the relations that define a machine as a unity, and determine the dynamics of interactions and transformations it may undergo as such a unity”, p. 16). By definition, the “structure” of a system constitutes the basic physical processes which realize it, which are undoubtedly determined by the laws of physics. But its “organization” precisely resides in the functional aspects of the relation between those processes which define the system as a biological unit and is thus constrained but not predetermined by the laws of physics. This distinction is critical to Varela’s [37] approach to life, as he defined biological autonomy as *organizational closure*—colloquially, the self-production of the organization. It is also critical to Varela et al.’s [38] approach to mind, as it is precisely the organizational closure of the cognitive agent which defines its co-productive relation with the world and thus enables them “laying down a path in walking” (Chapter 11) through engagement with the world. Thus, the enactive approach/biological autonomy builds from a *relational* definition of organization to understand the evolution of the (organizationally relevant) structure of life and mind—an approach which is constitutively in tension with a formalism which defines a system by its dynamical structure.

We argue in the present discussion that the FEP can nonetheless be formally reconciled with a relational definition of biological autonomy and even possibly ground it. We base this argument in Rosen’s [43,44] own approach to life and mind, which is similar in structure and spirit to Varela’s [37] although much more thoroughly formalized. Indeed, Rosen [44] provides a formal demonstration that an organism (defined as a system whose every component is itself produced from within) cannot be fully captured within what he calls the “Newtonian” approach, i.e., entailment from a single model assumed to describe its dynamics in full. This demonstration is based on Rosen’s [45] prior work on measurement theory, showing notably that the observable structure of a general dynamical system irreducibly depends on the relations in which it is embedded (barring a priori assumptions about its symmetries). We build on structural similarities between Rosen’s [45] formal approach and Fields, Friston, et al.’s [46] “quantum” formulation of the FEP to suggest that, understood in a specific formal context, the process of observation can be understood to *bring about* certain observables as “real” causal factors in the system’s activity—a property called *contextuality*. We further suggest that contextuality is adequately grounded in the thermodynamic symmetry breaking entailed by multiscale interaction within biological systems, building from Fields [47] and Fields and Glazebrook’s [48] prior work on the matter. On these grounds, we suggest that Fields, Friston, et al.’s [46] “quantum” FEP in fact constitutes the formal basis for a *relational* formulation of the FEP (rFEP), complementing its *classical* version (cFEP) by describing the self-constitution of biological organization and the following evolution in structure.

We further argue that this proposal is fully coherent with a naturalistic perspective on mind and life. Indeed, Beni [29] and Guénin--Carlut ([49], Chapter 2) propose what can likely be considered a common sense approach to what a formal framework can and cannot explain or represent, in the form of *structural realism* (SR). While it was developed by Worrall [50] to account for the continuity of intuition provided by successive formal theories of physics, structural realism affords a natural model of mathematical explanation, by which a mathematical framework “explains” whichever aspect of a system it does adequately capture [51,52]. This account alleviates pressure from realism arguments against both the cFEP and rFEP. Indeed, the cFEP may very well explain the emergence of intentionality (defined as the functional orientation of a system’s activity towards the world) from bare dynamics, even if those dynamics do not constitute a sufficient description of life and mind. Conversely, the rFEP may very well explain relational aspects of biological organization, even if its mathematics concerns the relational properties of organization rather than the underlying physical processes per se. Finally, we argue that the two can be understood as mutually coherent, complementary descriptions of Moreno and Mossio’s [53] thermodynamically grounded account of biological organization as a self-producing system of constraints. While the cFEP explains *synchronic* aspects of a system of constraints (i.e., symmetry-preserving activity within the dynamical structure entailed by a system of constraints), the rFEP explains its *diachronic* aspects (i.e., symmetry-breaking reconstruction of the system of constraints and the dynamical structure it entails).

In Section 2, we will expose how Rosen’s [43,44,45] formalization of the notions of observation and modeling cast doubt over the existence of a well-defined mathematical representation of life. In Section 3, we will discuss the thermodynamics of observation in biological systems and argue that the intrinsic cost of observation entails the emergence of *contextuality*, understood as the participatory production of the concepts deployed in cognitive engagement as “real states” of the agent–environment dynamics. In Section 4, we will explore how Fields, Friston, et al.’s [46] “relational” formulation of the FEP enables a formal treatment of contextuality based on observation with irreducible *reference frames* and its relation to Friston’s [6] “classical” formulation. In Section 5, we will address the epistemology and ontological issues raised by this participatory and relational approach to cognitive meaning. In Section 6, we will show how the broad framework we articulated throughout the present article relates to Varela’s [37] own notion of biological autonomy as organizational closure and more precisely its later reframing by Moreno and Mossio [53] and Montévil and Mossio [54] as a closure of constraints. Overall, we hope that this discussion demonstrates that the relational view of cognition is physically warranted, formally well-defined, and philosophically coherent. Most importantly, we show that it can intuitively be brought back to two fairly simple concepts: *contextuality* in cognitive engagement; and the description of cognitive organization in terms of *constraints* over the system’s activity, synchronically defining their intrinsic dynamics while diachronically being (re)constructed by their relational content.

## 2. Modeling Life?

Perhaps surprisingly, Rosen’s [43,44,45] program as we presented it above stems from a fairly mundane observation about the nature of scientific observation. By definition, when we *observe* a system, we do so through a specific physical interaction that enables us to retrieve some information about the state of this system. Conversely, the information we can effectively access in a given observation is mediated by the actual structure of this physical interaction. A meter can give us the length of a system but not its weight; a scale can give us the weight of a system but not its color; etc. Thus, any given mode of observation can only discriminate between certain states of a system, at the exclusion of others. Rosen ([45], Section 2.2) provides a fairly direct mathematical description of this. Let us assume a given observable is defined by the relation $f\left(S\right)=r_{m(s)}$; where *s* is the state of the measured system, *m* is the state of the measurement system, and *r* is the outcome of the measurement. By construction, an observable *f* can only discriminate between states up to equivalence classes ${\left(s\right)}_{R_{f}}$, each corresponding to the collection of states *s* which produce the outcome *r* under the observable *f* ([45], Definition 2.3.1). It follows directly that everything we could possibly observe about a system (directly or indirectly) is defined by a collection of such classes, corresponding to all the observables at our disposal. Thus, Rosen defines them as the *reduced states* of the system—they constitute by construction the system’s states, reduced to the space we can actually observe and manipulate.

On this basis, the classical intuition associated with what Rosen [43,44] later described as the “Newtonian paradigm” runs as follows. Given a careful study of the relation between the observables at our disposal, we should be able to identify a collection of (reduced) states which is necessary and sufficient to model the activity of the system and predict its evolution in time. Such a collection is defined by convention as the *state-space* of the system, and the rule by which variables evolve forward in time is defined as its *reduced dynamics.* (We should note that in physics and control theory, it is common to define dynamical systems as a differential equation and the *state-space* as the collection of base variables of the equation. In this context, the collection of necessary and sufficient variables to describe the system must include their relevant derivatives, and it is then called a *phase-space*. For the sake of clarity, we will use exclusively the mathematical convention in this article, where the state-space (by definition) includes all relevant variables). Then, the system can be fully described by a flow function (or law of motion) Φ:(T·X)→X, where *X* is the state-space and *T* is a time dimension. To ensure the coherence of the very concept of dynamics, Φ must further verify  Φ(0;x)=x (no movement occurs in the absence of time passing), $\Phi \left(t_{2};\Phi \left(t_{1};x\right)\right)=\Phi \left(t_{1}+t_{2};x\right)$ (the movement in $t_{1}$ then $t_{2}$ is equal to the movement in $t_{1}+t_{2}$), and $\Phi \left(t_{2}+t_{1};x\right)=\Phi \left(t_{1}+t_{2};x\right)$ (there is no path dependency in the time dimension itself). (It is technically possible to make coherent derivations in the absence of that last condition, as exemplified by Connes and Rovelli’s [55] thermal time theory. However, such approaches are quite rare even in fundamental mathematics, and when applied within physics they entail *extremely* exotic intuitions, associated, e.g., with Rovelli and Smolin’s [56] loop quantum gravity theory. They are fundamentally incompatible with any existing definition of the Newtonian paradigm—or even with any intuition coherent with our understanding of time). This defines the general mathematical framework of Dynamical Systems Theory (DST), which formalizes with maximal generality the notion of a system evolving along well-defined dynamics in a well-defined space and over a well-defined time dimension. (The reader could think of quantum physics as an exception to this paradigm due to the fundamental role of observation in bringing about quantum states. Nonetheless, the classical formalism of quantum mechanics—as was initially articulated by Born et al. [57] and later systematized by Dirac [58] and von Neumann [59]—explicitly assumes well-defined dynamics over the wave function of the system, which is itself defined over a well-defined state-space. Such wave functions just happen to be accessible to indirect measurement only and to be perturbed by observation. Thus, all relevant authors treat quantum mechanics either as a direct instantiation of the Newtonian paradigm or as a technical adjustment to it which remains insufficient to account for the complexities of mathematical biology). In this context, the evolution of the system is by construction fully entailed by its position in the state-space at a given point in time. Thus, assuming an adequate representation of the system’s state-space and dynamics, any deviation of the system from the trajectory predicted by our model given a measurement of its initial state must be due to an error or uncertainty in the measurement process.

However, Rosen [45] demonstrates that this intuition does not hold in the general case once we consider the constraints induced by the reduced description. In particular, let us consider the interaction between any two systems $S_{1}$ and $S_{2}$, assumed to be mediated by the mutual measurement of the collection of observables (*F*) and (*G*). On this basis, Rosen ([45], Section 3.6) derives that the general dynamics of interaction need not conserve the equivalence classes entailed by the respective collections of observables and need not be separable between the two systems. If an observer were to observe or model the dynamics of the composed systems in the state-space induced by (*F*) and (*G*), respectively, Rosen ([45], Section 3.5) outlines that the misalignment between their choice of representation and the actual dynamics of the system would appear as stochasticity or irreversibility. But, most importantly, a proportionate model of the composed dynamics would necessitate the use of a novel state-space defined by novel observables, with no apparent roots in the state-space of either system prior to interaction. This would appear to the naive observer as the mysterious emergence of entirely novel structure in the composed system, although no structure is added beyond the interaction ([45], Section 4.5). In other words, even a well-defined state-space representation and associated reduced dynamics only entail a coherent description in a specific, *closed* regime in the general case. Once the system is *open* to interaction, the assumptions defining that regime cease to hold—even if the world our system is embedded in accepts itself a well-defined state-space representation and associated reduced dynamics.

Hence, Rosen ([45], Section 3.1) concludes that the representation of a system’s dynamics as a well-defined law of motion embedded in a well-defined state-space cannot be derived from observation alone. (Although Rosen [43,44,45] falls short of making that statement, it arguably constitutes his overarching philosophical thesis—to the extent there is one. Indeed, his entire development in Rosen [45] serves to demonstrate that the relational properties he later studied in Rosen [43,44] emerge from the study of systems equipped with well-defined but unobservable dynamics, given the assumption that such dynamics should be described only in terms of specific collections of observables and their relations.) Rather, the formulation of laws of motion must rely on additional (implicit or explicit) assumptions. Namely, those assumptions concern the *symmetries* of a system—as per Rosen ([45], Sections 6.5 and 6.6), a group of transformations over the system’s states which (by construction) does not affect its (reduced) dynamics in a given representation. This statement in itself is not particularly novel, as the profound duality between physical symmetries and laws of motion has been the basis for most mathematical physics since Noether’s [60] seminal work. However, Rosen ([45], Chapter 4) demonstrates that the symmetries of any given system can in principle be established or broken by its general dynamics. (This statement is somewhat unfaithful to Rosen’s ([45], Chapter 4) argument, which is framed in terms of *linkage* rather than *symmetries*. However, Rosen repeatedly accentuates throughout the book that the two perspectives are dual. In particular, Rosen ([45], Sections 6.3 and 6.5) establishes that symmetries entail an equivalence which itself constitutes a linkage, so Rosen’s ([45], Chapter 4) argument extends to the concept of symmetry per se. Thus, we prefer to express the underlying argument in the language of symmetries, which is both more familiar in mathematical physics and more relevant to our discussion). This is especially true (as we have seen in the previous paragraph) when they are brought into interaction with the broader world. Thus, any representation of a system’s dynamics by a given law of motion can only hold as long as the relevant symmetries apply. This principle can be illustrated by the trivial case of a pendulum. One would readily agree that the latter accepts a well-defined law of motion, defined by the action of gravity and the reaction force from the string carrying it. But, were one to introduce an unforeseen interaction in the system (e.g., friction progressively damaging the string), some new facts about the system would become causally relevant, and its laws of motion should be rewritten accordingly (e.g., accounting for its eventual rupture).

However, the core contribution of the book consists in Rosen’s ([45], Chapter 5) mathematical analysis of the modeling relation in and of itself. Building from the discussion we summarized above, he defines the relation between a system *S* and a model thereof *M* as the equivalence between their dynamics, as reduced, respectively, over the collections of observables ($F_{S}$) and ($F_{M}$) in Rosen ([45], Section 5.1). This treatment enables Rosen ([45], Section 5.2) to provide an explicit mathematical account of abstraction in modeling, formalizing the common insight that models work by abstracting contextually irrelevant features of the target system (see, e.g., [61,62]). But, most importantly, it enables a rigorous formal treatment of how different models, relying on different abstractions (and thus different collections of observables and assumed symmetries) may interact to capture the dynamics of a given system. This is what defines, in Rosen’s ([45], Section 5.7) view, his relational approach, as it provides a basis to study the dynamics of any given system *in relation to* the class of systems which models it. Formally speaking, this approach is based on category theory, a highly abstract mathematical theory which describes mathematical structure itself based on the construction of correspondence between the space of objects and relations defined by theories (i.e., categories). This formal approach, while rigorous, is well beyond what we can synthesize accessibly in the present discussion. Thus, we will focus explicitly on the conceptual implications of Rosen’s [43,44] later work, which relies explicitly on the category-theoretical description of the modeling relation he motivated in Rosen [45].

Rosen [43] is primarily concerned with the formalization of its eponymous concept, *anticipatory systems*. Rosen ([43], Foreword) defines an anticipatory system as “one in which present change of state depends upon future circumstances, rather than merely on the present or past”, which he notes stands in direct conflict with the Newtonian paradigm. To resolve that conflict, Rosen ([43], Chapter 4) proposes a formal treatment of time-encoding as a participatory dimension entailed by the irreversibility of dynamics within a system (given a specific collection of observables). On this basis, Rosen ([43], Chapter 6) describes future-dependency as emerging from an internal model of one’s world, whose time-encoding happens to be faster than that of the world it engages with. But, most importantly, Rosen ([43], Chapter 5) demonstrates that the abstraction required by the modeling relation can only hold under limited conditions (Sections 5.4 and 5.5), and that deviation from those conditions will appear to the anticipatory system as the emergence of novel observables (Section 5.9). The conceptual analogy to Friston’s [1,3,6] FEP would be hard to miss, but we must clarify that the two formalisms are motivated by fundamentally incompatible orientations and assumptions. Indeed, Rosen *assumes* the existence of the modeling relation to *explain* the dynamics of engagement with the world—the exact opposite of Friston’s construction. But, most importantly, Rosen’s [43] formalism is explicitly based on a relational conception of the relation between mind and world: what matters causally to the agent’s behavior is the space of observables defined by the modeling relation it embodies, and this space is by construction incompatible with a dynamical representation.

In doing so, Rosen [44] articulates what is generally accepted as the canonical formulation of relational biology. Rosen ([44], Prolegomenon) first argues that the domain of mechanisms (corresponding to the Newtonian paradigm) in fact constitutes a very small region of the space of all possible systems. To formalize this idea, Rosen ([44], Chapter 8) defines a mechanism as a system which only admits simulable models (which can be computed on a Turing machine) and demonstrates that such systems must admit a unique maximal model defined as the sum of its minimal models. Intuitively, this entails that a mechanism accepts a well-defined representation as the composition of its subsystems, which fully entails its activity (Sections 8G and 9D). Rosen ([44], Chapter 9) further derives that a mechanism cannot verify *closure to efficient causation* (Sections 9E and 9F): as its material organization is fully entailed by its maximal model, it must be defined prior to and away from its own activity. By contraposition, Rosen ([44], Chapter 10) defines an organism as a system closed to efficient causation (i.e., which produces its own material organization) and derives that such a system cannot accept a single largest model. Rosen ([44], Sections 10C and 10D) further formalizes the mathematical structure of self-production as a (M, R)-system (composing metabolism and repair) and discusses how this relational description of self-production describes the evolution of structure in biological systems ([44], Chapter 11). This framework, together with the negative result that (within his specific formal construction) an organism cannot be fully represented within a given model, constitutes the most influential and largely cited aspect of Rosen’s overall work.

At face value, Rosen’s [43,44,45] approach appears to undermine the very coherence of the physics-based approach to cognition proposed by Friston’s [1,3,6] FEP. Indeed, the FEP is by construction a dynamical systems-theoretic approach to life and mind, and Dynamical Systems Theory is by construction the natural formalization of the Newtonian paradigm rejected by Rosen [43,44,45]. However, this does not entail that a formal, model-based approach to life and mind cannot provide another form of explanatory value: for example, building some form of physical intuition regarding the relational processes which Rosen describes only through existence and non-existence theorems. Thus, we would rather recruit Rosen [44] as a thorough, formally well-defined formulation of what a relational approach to life and mind entails exactly and what it enables. At its core, the relational approach can be brought back to one proposition: the space in which a system’s activity can be mathematically embedded is fundamentally defined in relation to the specific (internal or external) physical interactions it is engaged in. More specifically, Rosen [45] formalizes this through the role of *observables* in measurement, on the basis of which Rosen [43,44] attempts to articulate a coherent relational theory of the evolution of structure in the (reduced) dynamics of mind and life. But, critically, this theory is uniquely centered on the description of biological organization and its relation to the underlying dynamical structure, rather than describing the physical principles by which this organization comes to be. To bridge that gap, we will attempt to reframe the relational approach from a basic thermodynamical approach to the role of observation in biological systems.

## 3. The Thermodynamics of Observation in Biological Systems

At a conceptual level, the notion of *observation* is intrinsically intentional: an observation is a process by which a physical system comes to learn something about the world. Nonetheless, it is bound by well-studied physical principles, stemming from Clausius’ [63], Boltzmann’s [64,65], and Gibbs’ [66] early formalization of statistical mechanics. Collectively, they provided the basis for what is known today as the second law of thermodynamics, stating that the *entropy* of an isolated system cannot diminish. (Our contemporary understanding of the second law is grounded in the later derivation of Evans and Searles’ [67,68] and Gallavotti and Cohen’s [69,70] fluctuation theorems, which establish on a rigorous far-from-equilibrium basis that although negative entropy fluctuations can indeed occur at the microscopic scale, exponential cut-off makes such fluctuations statistically insignificant at a macroscopic scale). A full discussion of the complex history of the second law of thermodynamics is beyond the scope of this paper, lest we risk sharing Boltzmann’s tragic fate. (A paraphrase of Goodstein’s [71] famous witticism in his handbook on the topic: «Ludwig Boltzmann, who spent much of his life studying statistical mechanics, died in 1906, by his own hand. Paul Ehrenfest, carrying on the work, died similarly in 1933. Now it is our turn to study statistical mechanics. Perhaps it will be wise to approach the subject cautiously.» (p. 1)). Nonetheless, we should note that this principle is generally considered to constitute one of the single most fundamental structures in physics. Notably, it constitutes the most widely accepted explanation for the symmetry breaking which enables the very emergence of time as an irreversible dimension, despite the time symmetry of physical laws [72] (see also [43], Chapter 4). The second law can be written as follows for a process taking an arbitrary system from macro-state A to macro-state B, assuming a micro-state noted μ:

$$\underset{A\to B}{dS}⩾0;\;\mathrm{where}S=-k_{B}\cdot \int_{\mu }p\left(\mu \right)\cdot \ln p\left(\mu \right)\cdot d\mu =k_{B}\cdot I\left[p\left(\mu \right)\right]$$

Jaynes [73,74] noted the formal connection between the thermodynamic and information-theoretic concepts of entropy, as visible in the formula above. The thermodynamic entropy *S* of a system indeed corresponds (up to Boltzmann’s constant $k_{B}$) to the information-theoretic entropy *I* of the distribution over its microstates, given the state of its macroscopic observables.

On the basis of this connection, we can investigate the thermodynamic basis of the process by which an agent observes the world they engage with. Assuming that a subject *S* measures some aspect of an object *O* so as to gain *n* bits of information about it, the entropy of the joint system (S,O) would diminish by a corresponding amount $\underset{A\to B}{dS}=-n\cdot k_{B}\cdot \ln\left(2\right)$ as the internal states of *S* evolve to reflect the outcome of the measurement about *O*. If (S,O) constitutes a closed system, this is of course impossible by the second law of thermodynamics. Thus, the measurement of *n* bits must necessarily produce an equal or superior quantity of entropy $s_{A\to B}$ to respect the second law: $\underset{A\to B}{dS}=s_{A\to B}-n\cdot k_{B}\cdot \ln\left(2\right)⩾0$. By construction, this means the measurement process must *destroy* at least as much information as it uncovered—either by exhausting this entropy into the object *O*, thus erasing at least as much information as was observed, or by exhausting this entropy into the subject *S*, thus forgetting at least as much information as was observed, or any combination of the two. Thus, it appears that observation is a zero- or negative-sum game. While subjects may learn about novel aspects of the world by observation, any given observation can only conserve or increase the amount of uncertainty they have about the world. Of course, this is an absurd conclusion: empirically, it appears beyond reasonable doubt that we can indeed resolve uncertainty about the world by observing it. Thus, it must be that measurement does not occur in an (S,O) system taken in isolation.

The classical formal treatment of the role of the environment in the physical process of observation follows from Landauer’s [75] principle. In substance, Landauer’s principle states that any logically irreversible operation entails the dissipation of a corresponding amount of heat $h_{A\to B}=s_{A\to B}\cdot T⩾n\cdot k_{B}\cdot T\cdot \ln\left(2\right)$ into the environment. Under this premise, we can understand the thermodynamic cost of observation on the basis of an (S,O,E) partition where *O* encompasses the states of the world being measured, *S* encompasses the states of the world which enact the measurement, and *E* encompasses all degrees of freedom of the world that are irrelevant to observation. Thus, it follows that the successful measurement of *n* bits in *O* by *S* (where “success” supposes that uncertainty resolved by observation is not immediately reinjected in information-bearing degrees of freedom) entails the exhaustion of $s_{A\to B}⩾n\cdot k_{B}\cdot \ln\left(2\right)$ into *E*. As *S* is by necessity a physically embedded system with a finite number of degrees of freedom, it cannot retain the exact memory of an infinite number of measurements. Thus, when the system inevitably makes room for a new observation by deleting *n* bits of information, Landauer’s principle assimilates the exhaustion of $s_{A\to B}$ into *E* with the dissipation of a corresponding amount of heat $h_{A\to B}=s_{A\to B}\cdot T⩾n\cdot k_{B}\cdot T\cdot \ln\left(2\right)$. Importantly, this argument applies to *observation*—the process by which the agent imprints information-bearing degrees of freedom about the world on its own information-bearing degrees of freedom—but also to *computation*—construed broadly, the process by which the agent’s internal dynamics then propagates and integrates this information over time.

However, this reasoning relies on critical assumptions that do not necessarily apply to any given instance of observation or computation. As discussed in Bennett [76,77], the dissipation of excess entropy as heat is specifically the outcome of a logically irreversible operation in the process of computation or measurement, such as the merging of distinct computational threads, the deletion of memory, or the resetting of a measurement apparatus. More specifically, Bennett [76,77] assumes a cyclical process of preparation and measurement that we will note as M∘P. (The order here is intentional. ∘ denotes the composition of two operations, and M∘P thus denotes the application of the operation *P* (prepare) and then only the application of the operation *M* (measure)). *M* constitutes the measurement itself, understood as the copy of the measurement outcome into the measuring system. *P* constitutes in turn the preparation, understood as the reinitialization of the measuring system into a neutral state. In this context, *M* copies *n* bits of information corresponding to the outcome of the measurement. This is in itself a reversible process, as it takes the system from a deterministic state (S=0) to another deterministic state (S=0) entailed by the outcome of measurement. Hence, computing architectures which circumvent the occurrence of an irreversible preparation step avoid altogether the generation of heat by Landauer’s principle—as established empirically, e.g., by Orlov et al. [78] or Dago and Bellon’s [79] study of such reversible architectures. Much in the same manner, observation processes which circumvent the occurrence of a logically irreversible step do not entail heat generation by Landauer’s principle. Thus, we cannot assume that the biological processes we are studying here can or should be expected to exhaust as heat the excess entropy entailed by the observations or computations they enact. (See also Norton’s [80,81,82] argument that Landauer’s [75] principle and its later refinement by Bennett [76,77] both presuppose rather than demonstrate the identity between logically and thermodynamically irreversible processes and thus fail to establish a mechanism for heat generation in logically irreversible processes—and, by extension, they fail to establish Landauer’s principle as a physical law).

Indeed, both Landauer [75] and Bennett [76,77] assume a clean separation between information-bearing and non-information-bearing degrees of freedom. This reasoning applies well enough to human-made computational systems implementing Turing’s [83] architecture, as they ultimately function through the manipulation of an array of binary states implemented in a magnetic tape or a semiconductor storage placed at a specific point of the architecture—thus structurally making the material implementation of those binary states the information-bearing degrees of freedom, to the exclusion of any other degree of freedom the system may have. Thus, as noted by Rosen [44], their activity can be adequately described by the reduced dynamics within the state-space defined by their information-bearing degrees of freedom. To borrow Varela’s [37] terminology, their *organization* (understood as the relational system of processes which entail their definition as systems) and their *structure* (the law of motion defining their dynamics in the full state-space) happen to coincide. But, if we recall our discussion in Section 2, this is precisely the property which Rosen denies in the case of biological systems. In Rosen’s [43,44,45] framework, it is precisely the process of observation which brings about physical states as organizationally significant drivers of the system’s activity, i.e., observables. The question we must address is therefore what physical sense (if any) we can make of a process of observation premised on a relationally defined organization rather than a system defined with an intrinsic state-space—and, more specifically, what sense we can make of entropy and its flux within this relational framework.

At a conceptual level, we may find some support in an unexpected place: Tegmark’s [84] investigation of the entropic problem in quantum cosmology. While the questions Tegmark [84] tackles fall well outside the scope of our discussion, he does provide a rigorous formal account of entropy flux within an (S,O,E) partition much like our own—and under the explicit definition of entropy as an observer-dependent quantity. In this account, entropy conservation over the SOE system is enabled by the opposite effects of the *S*–*O* interaction (i.e., observation, reducing entropy relative to the subject) and the *E*–*O* interaction (i.e., decoherence, augmenting entropy relative to the subject). To be clear, both interactions are understood to be driven by the entanglement between the states of *S* (resp. *E*) and those of *O*—a specifically quantum mechanism with no known implementation in biological computation given rapid decoherence time in biological settings [85]. Nonetheless, we may consider the question of whether the entropy flux entailed by the observation of physical states within the context of biological organization produces analogous effects. For example, Pessoa [86] suggests that neurocomputational function emerges from “entangled” representations in the classical sense that their activity is embedded across brain regions without dynamical separability between subsystems or subprocesses. While this argument is in itself fully coherent with a Newtonian intuition, it partially reproduces Rosen’s [44] insight that organizationally relevant facts about the physical states of a biological system are defined in relation to their observation—understood here, in Rosen’s [45] sense, as the broad array of processes which makes them organizationally relevant in the first place.

Fortunately, this specific argument can be derived formally within a fully classical framework through a careful, explicit account of the thermodynamic cost of *identifying what is being measured*. Indeed, Fields [47] and Fields and Glazebrook [48] investigated the effects of discriminating the system of interest *O* against non-informative aspects of the world *E* (given finite resources). By construction, system discrimination entails a symmetry breaking between the states of *O* (treated as informative in the context of the measurement process) and the states of *E* (treated as the background for and fuel of the observation). On this basis, Fields [47] and Fields and Glazebrook [48] demonstrated that this symmetry breaking must be irreversible and must entail the breakdown of commutativity between successive identification-and-measurement operations—at least when those operations are defined over all degrees of freedom of (S,O,E) rather than a coarse-grained state-space corresponding to the measurement basis. The full formal basis of this argument is beyond what we could articulate accessibly in the present discussion, but its physical meaning is rather simple. Indeed, non-commutativity in measurement operations, or *contextuality*, has long been known to be incompatible with the existence of a sufficient model of a system through its reduced dynamics sensu Rosen [44,87,88,89,90]. In other words, Fields [47] and Fields and Glazebrook [48] demonstrate that the thermodynamic cost and the symmetry breaking induced by measurement must irreversibly *bring about* the observables it measures as constitutive states of the system’s reduced dynamics.

Of course, the fact that this result holds mathematically does not entail that it is relevant to the study of any given system. For example, classical computation is a deterministic process by construction in the absence of bugs. However, this can be readily explained by the fact that system identification is trivial in the case of computers due to their mechanistic nature, sensu Rosen [44]. The state being measured at any time step is by construction the data encoded in the system memory at the previous time step. Thus, any symmetry breaking between informative and uninformative states must occur either before or after the computation per se—during the encoding of the software and data and the decoding of the computation outcome. However, and as we discussed above, biological systems are very much unlike computers in this regard. The computations they perform are by construction embedded within multiscale, reversible dynamics spanning virtually all accessible physical degrees of freedom [91,92,93]. Thus, the process of deciding which degrees of freedom are relevant for regulating what processes cannot be abstracted away for biological computation in the general case. In other words, Fields’ [47] and Fields and Glazebrook’s [48] argument provides the basis for a physically grounded, relational theory of life and mind by formalizing a classical mechanism for the emergence of contextuality in biology and cognition. In the next section, we will discuss how this relates to the coherence of the FEP as a general theory of biology and cognition.

## 4. A Tale of Two Free Energy Principles

As is generally accepted even by its critics, the core contribution of the FEP is to provide a formally grounded description of the intentional properties of mind and life, based uniquely on the representation of the underlying dynamics. This is in itself a great achievement, precisely because (as we have seen in Section 2 of the present article) Dynamical Systems Theory constitutes the general formalization of the Newtonian paradigm. In particular, it is trivial to derive that DST is structurally incompatible with the property of contextuality, defined above as the non-commutativity of observation operators. Let us describe the interaction between two systems as the law of motion Φ:(T·X)→X. For any two evolution operators $\Phi_{1}:x\to \Phi \left(t_{1};\circ \right)$ and $\Phi_{2}:\Phi \left(t_{2};\circ \right)$:

$$\Phi_{1}\cdot \Phi_{2}=\Phi \left(t_{1};\Phi \left(t_{2};\circ \right)\right)=\Phi \left(t_{1}+t_{2};\circ \right)\underset{!\;t_{1}+t_{2}=t_{2}+t_{1}}{=}\Phi \left(t_{2}+t_{1};\circ \right)=\Phi \left(t_{2};\Phi \left(t_{1};\circ \right)\right)=\Phi_{2}\cdot \Phi_{1}$$

The same result holds by extension for the projection of the flow function on any partition of *X*; thus, all interactions between subparts must commute, and none can be taken to represent a contextual process of observation of a system by another. This is a direct implication of the intuition of a system following a well-defined flow along a well-defined time dimension. (We should note that additional structure can be defined over this definition, which may itself be non-commutative. Our discussion of Rosen in Section 2 provides an example of such a structure. However, the non-commutativity is then a property of the additional structure we have postulated and thus requires specific attention regarding its own conceptual presuppositions and implications. This approach is exactly what we attempt to do here via the formal grounding of observables in Fields’ approach to physical observation). We should note that this result does not hold if we allow for the definition of a non-commutative time dimension (such that $t_{1}+t_{2}=t_{2}+t_{1}$ does not hold in the general case). While technically well defined, this approach translates the *extremely* exotic notion of time as a branching, path-dependent dimension of variation. (This approach was proposed independently by Rosen ([43], Chapter 4) and Connes and Rovelli [55] to reconstruct the concept of time itself from thermodynamic irreversibility. Both are explicitly working within a relational approach, incompatible with the very premise of describing natural system through their reduced dynamics). It supports neither the FEP’s ambition of explaining the intentional properties of mind and life from simple, physically well-defined dynamics nor the definition of basic operations such as derivation in time, on which the FEP explicitly relies.

Thus, we will consider instead the alternative formulation of the FEP proposed by Fields, Friston, et al. [46] (see also Fields, Glazebrook, et al., [94]; Fields et al. [95]; Fields and Glazebrook [96]; Fields et al. [97]), on the basis of the derivation from Fields [47] and Fields and Glazebrook [48] we discussed in the section above. Here, again, we will not be able to articulate the full logic of their argument due to its heavy reliance on the category theory-adjacent framework of Barwise and Seligman’s [98] Channel Theory. This approach provides a theory of information flows in complex systems by reconstructing the Shannonian notion of information channels. Thus, it allows Fields, Friston, et al. [46] to reframe the transfer of information across a Markov blanket as a classical Shannonian information channel encoding the output of a contextual observation. Two important nuances distinguish this approach from the classical formulation of the FEP (hereafter, the cFEP) by Friston [1,3,6]. First, the locus of interaction is defined as a holographic screen, i.e., a virtual boundary defined by the information transmitted across it. This is in contrast to the cFEP’s definition of the Markov blanket as a section of the pre-existing state-space, which happens to verify conditional independence of the attracting distribution entailed by the system’s dynamics. Second, the process of observation is held to be contextual. Thus, the information transmitted through the Markov blanket/holographic screen is a non-trivial product of the observation basis or *reference frame* developed by the agent system. As we will argue, those properties equip Fields, Friston, et al.’s [46] formalism with the adequate structure to be considered a relational reframing of the FEP (hereafter, the rFEP).

An important aspect of the rFEP is the description of the observation process within a von Neumann algebra [59], which constitutes by construction the simplest coherent mathematical structure capable of supporting contextual observation as the action of matrix calculus. We should note that it also constitutes the mathematical basis of the canonical formulation of quantum mechanics by Born et al. [57]. We will discuss in more detail the epistemological implications of that statement in the following section, but we must first qualify a few disambiguations. Most importantly, and even though Fields, Friston, et al. [46] frame their approach as formulating an “FEP for quantum systems”, nothing in their derivation assumes the underlying physics of the system to belong to the quantum realm in and of itself. Where Fields, Friston, et al. [46] call on entanglement (the one property generally thought of as the marker of the quantum realm), it describes specifically the non-separability between the reference frames driving the observation, rather than between ontic states of the system. But, if we recall our discussion of Rosen [43,44,45] in Section 2, the non-separability between the observation processes entailed by various physical interactions in a system is the direct product of a relational perspective on its organization. (We should note here that Rosen can hardly be suspected of smuggling quantum mechanical assumptions in his mathematics, as he repeatedly contests the non-realism entailed by Born et al.’s [57] approach throughout Rosen [45]). And, as we have discussed in Section 3, the entropy flow intrinsic to perception can itself lead to the entanglement (in the technical sense of non-separability) of cognitive function across a distributed system, as was formalized by Fields [47] and Fields and Glazebrook [48] on the basis of a fully thermodynamical mechanism.

Thus, while Fields, Friston, et al. [46] frame their argument as a formulation of the FEP for generic quantum systems, it can just as well be considered as a physics-based reconstruction of Rosen’s [45] mathematical intuition about measurement. In doing so, it motivates a significantly different physical intuition than Friston [1,3,6]. The rFEP indeed redefines variational free energy (VFE) as the prediction error over the content of the information channels entailed by an observation, which is by construction contextually brought about by a specific reference frame. Thus, VFE can be rewritten as the sum of the intrinsic uncertainty induced by the misalignment between the reference frames, respectively, deployed by the agent and the environment (similarly to Heisenberg’s [99] uncertainty principle) and the classical inaccuracy induced by insufficient learning. Then, Active Inference emerges naturally as the reduction of VFE induced by the repeated preparation and measurement of the world across the boundary within a given reference frame. In this context, VFE can converge towards zero *if and only if* the agent and its environment converge towards non-separability, meaning that no information can be transferred between them as they effectively become the same system. This entails that, in this formulation, self-evidencing is to some extent antagonistic to the full relaxation of the system. To keep existing as a distinct system, an agent must actively maintain its boundary as a non-trivial interface with its environment, characterized by its capacity to function as a classical information channel. This process can only happen at a non-trivial thermodynamic and informational cost, and (by definition) it must necessarily bring about the observables defined by the agent’s reference frame as irreducible causal states of the boundary space. (We will note that Friston’s [6] formulation of the FEP describes inference as the minimization of the VFE associated with the distribution q(e∣λ)∼p(e∣b,λ), which concerns by construction the pre-existing, intrinsic states of the environment. Furthermore, the (Markov) boundary its formulation relies on is entailed by the attracting distribution of the system p(Λ,B,E), which is assumed to be well defined. Thus, it is itself a structural property of the a priori description of the system at relaxation, not an organizationally precarious and thermodynamically costly product of the system’s own activity away from relaxation. The same holds for Friston et al.’s [100] path integral formulation of the FEP given its common reliance on the same dynamical system-theoretic definition [101,102]).

To explain in more detail the distinction between the two, we will here again describe these two approaches from the role they provide to physical symmetries. As we have discussed in Section 2 of the present article, most of mathematical physics is framed in terms of *symmetry groups*: closed, associative families of transformations over a system’s state-space which do not affect its dynamics and, in most cases, define the structure of a theory. For example, special relativity is defined by the conservation of the speed of light between different (inertial) reference frames, entailing the symmetry of its physical laws with regard to the Lorentz transformation group [103] and thus the familiar geometry of spacetime. A special case emerges in the case where the symmetry group is a continuous space of transformations affecting each point of the system independently (a Lie group). Indeed, such structures entail a non-trivial geometry shaping the trajectories of systems, which appear to be corrected by force-carrying fields enforcing the underlying symmetries against perturbations. A direct illustration of this is the manner in which the geometry of space-time under general relativity (which, unlike special relativity, relies on a local symmetry) appears to us as a “gravitational force”, a physical field pulling massive objects together. The study of such structures is named *gauge theory*, and its logic underlies the canonical physical description of all fundamental forces [104]. (We should note that gravitation, while admitting a gauge-theoretic description in the form of general relativity, remains the only fundamental force that was not successfully *quantized*—that is, equipped with a corresponding force-carrying particle and the associated quantum field description). Incidentally, Sengupta and Friston [105] and Sengupta et al. [106] have discussed an alternative, gauge-theoretic formulation of the cFEP where symmetries in the system’s Lagrangian (by construction, this corresponds to the expected free energy later introduced in Friston et al.’s [100] path integral formulation) are expressed in the system’s observable dynamics through the effect of precision weighting.

**Figure 1:**
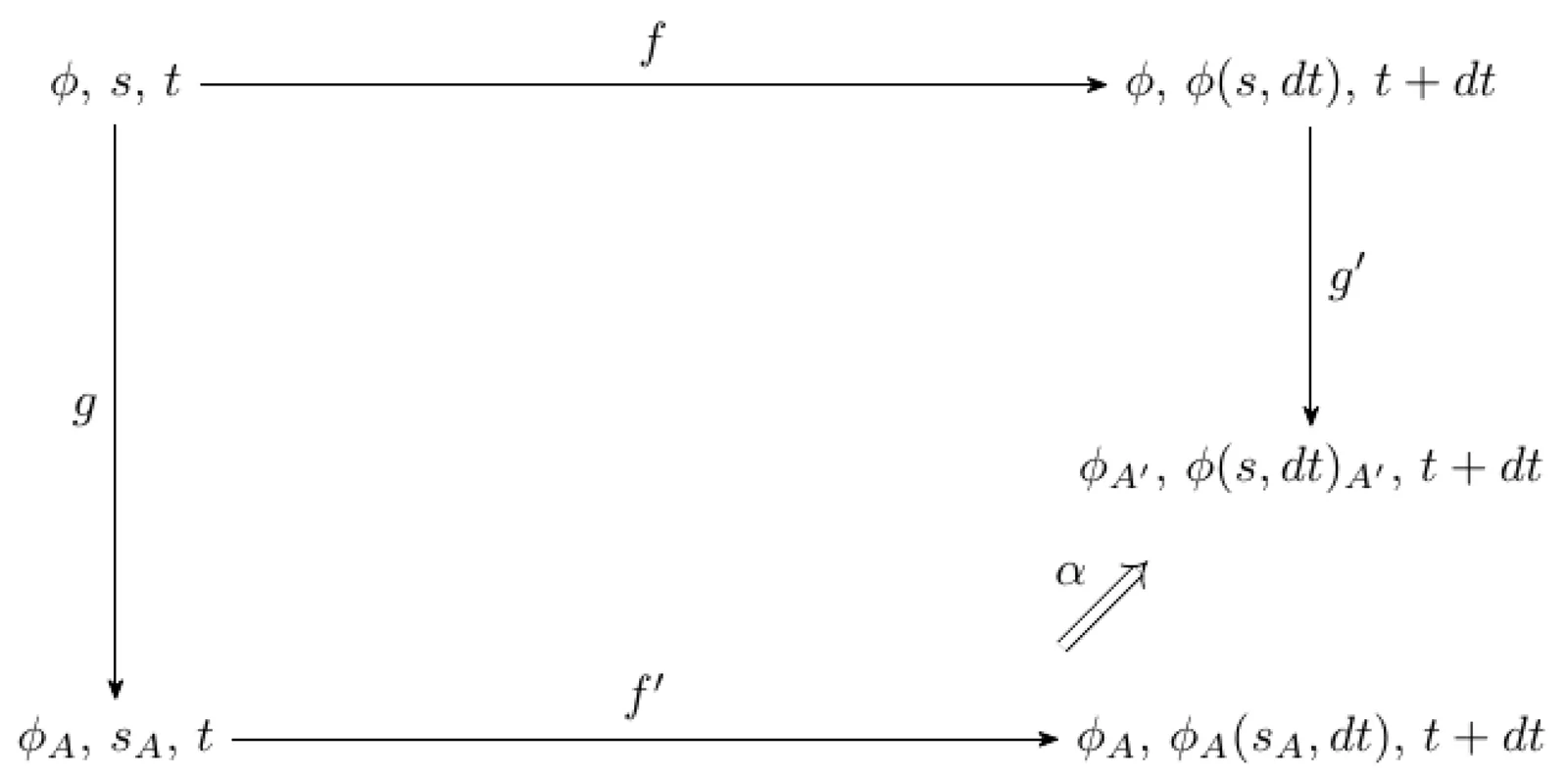
A diagrammatic representation of the proposed relation between cFEP and rFEP. The open square diagram above is intended as an intuitive diagrammatic representation of the relation between cFEP and rFEP we have described just above. The operation *f* (resp. $f^{\prime }$) corresponds to the evolution of the system following the flow ϕ (resp. $\phi^{\prime }$) for a time dt, as prescribed by the cFEP. The operation *g* (resp. $g^{\prime }$) corresponds to the observation of an observable *A* (resp. $A^{\prime }$), as prescribed by the rFEP. Most importantly, this diagram illustrates that “evolution” under the cFEP and the rFEP, respectively, constitutes two fundamentally different kinds of operations. While *f* (resp. $f^{\prime }$) constitutes evolution within a well-defined space *S* (resp. $S^{\prime }$) entailed by the well-defined flow function ϕ (resp. $\phi^{\prime }$) over a time step dt, *g* (resp. $g^{\prime }$) constitutes the transformation of the system (ϕ,S,T) into $\left(\phi_{A},S_{A},T\right)$ (resp. $\left(\phi_{A^{\prime }},S_{A^{\prime }},T\right)$) entailed by the novel symmetries produced by the observation of *A* (resp. $A^{\prime }$). Moreover, we should note that, self-evidently, the observations conducted by a system should depend on its state in the relevant reduced space at a given time. Then, the reduced states and dynamics of the systems defined by the two paths $f^{\prime }\circ g\left(\phi ,s,t\right)=\phi_{A},\phi_{A}\left(s_{A},dt\right),t+dt$ and $g^{\prime }\circ f\left(\phi ,s,t\right)=\phi_{A^{\prime }},\phi {\left(s,dt\right)}_{A^{\prime }},t+dt$ should remain irreducibly different in the general case. The arrow α is the canonical diagrammatic representation of this difference, defined so that $\alpha \left(f^{\prime }\circ g\right)=g^{\prime }\circ f$. The study of its properties (and of the conditions under which it is well defined) would be an important first step in an integrated formalization of the cFEP and the rFEP, which we do not attempt to conduct here. cFEP: classical Free Energy Principle; rFEP: relational Free Energy Principle.

While symmetries are generally conceptualized as intrinsic, structural properties of a theory, we should nonetheless point out with Rosen ([45], p. 137) that they are defined as a group of transformations which is “visible in one description of a system” (usually assumed to be the generalized state-space in which a system is defined) “but not in another” (usually assumed to be the reduced state-space on which its observable dynamics are defined). In other words, a statement about symmetries is a statement about the relation between two descriptions of a system, defining which facts or structural elements are observable in one but not another. On this basis, we can observe that the cFEP—as understood through the gauge-theoretic framing of Sengupta and Friston [105] and Sengupta et al. [106]—and the rFEP describe two different and complementary aspects of the organization of complex systems. On one hand, the cFEP describes how *the symmetries of a system* (as defined in the reduced state-space where its observable dynamics are embedded) bring about its activity and, more specifically, defines *the observations it will make about the world* (the generative model it enacts). Conversely, the rFEP describes how *the observations a system makes about the world* (the reference frames it mobilizes) bring about its symmetries (as defined over its general dynamics, sensu Rosen). In other words, the cFEP captures the organization of meaning entailed by reduced dynamics of a system within a reduced space, while the rFEP captures the evolution in the reduced space and dynamics produced by the organization of meaning of a system (Figure 1).

The relevance and complementarity of those two perspectives should appear naturally when we consider their relation to Friston, Fagerholm, et al.’s [107] renormalization approach to the cFEP. This approach starts from a detailed description of the system’s dynamics and iteratively coarse-grains it by abstracting away the faster dynamics at a given scale. This enables the cartography of the architecture of hierarchical Bayesian inference from empirical data, although it relies on an instrumental, transient definition of Markov blankets (where the cFEP may or may not apply). However, this does very little to explain how the structure thus inferred may evolve over time, given the underlying assumption of a fixed dynamical structure. Instead, we can recruit the rFEP to describe the synchronization between elements of the system at any given scale as the construction of a shared communication channel, produced by the distributed application of a given reference frame to their shared biological medium. The non-commutativity of this operation emerges naturally from the thermodynamical irreversibility of the choice of reference frame, and the non-separability of their reference frames constitutes in turn a natural information-theoretic expression of what we describe in dynamical systems-theoretic terms as synchronization. Applied at nested scales, this description reproduces the notion of biological organization as a process of hierarchical Bayesian inference. But, importantly, it equips this architecture with the ability to *bring about* the novel observables entailed by their downward predictions as constitutive states of their reduced dynamics, thus non-trivially (re)producing their constitutive symmetries.

The notion of unfolding symmetries should be quite easy to accept, at least in the context of biological organization. However, the notion that their evolution is defined by meta-causal “reference frames” which define what they can or cannot observe about the world is less immediate. To define and defend the call on reference frames, Fields and Levin [108] define information as what “makes a difference for what an organism can do” (Section 3). Within the framing of the cFEP, this corresponds directly to the states of a system’s Markov blanket which have a measurable effect on its internal dynamics. A very classic example would be bacterial chemotaxis, which suggests that bacteria can perceive certain chemical states as |good> or |bad>, as operationalized by their direct sensori-motor coupling. This in itself can very well be described within the cFEP in purely causal-dynamical terms. But, if we consider in detail the chemical composition of this environment, we would be hard pressed to measure the chemical concentrations of |good> and |bad>, and the bacteria would find it just as hard to describe the full micro-states of solution they experience as such. It would follow that the states of a Markov blanket, to the extent that they are what informs an organism’s response to the world, are (at least in part) defined by the selective openness to the world entailed by their constitutive organization. Before we turn to discussing the implications of such an organizational account of mind and life, we will investigate the philosophical bearings of the idea that natural systems can produce their interface with the world.

## 5. Being Realist About Contextuality

At this point in our discussion, the reader will likely have caught on to the argument we were aiming to make. If they recall the distinction made in the introduction between *synchronicity* (the study of meaning within a given structure) and *diachronicity* (the study of evolution in such structure), they will notice that the two perspectives correspond quite precisely to the formal approaches proposed, respectively, by the cFEP and the rFEP. While Friston’s [1,3,6] cFEP describes the flow of cognitive engagement within a given (dynamical systems-theoretic) structure, Fields, Friston, et al.’s [46] rFEP describes the evolution of the structure underlying cognitive engagement given its (information-theoretic) relation with the world. We cannot emphasize enough that this complementarity is not incidental but is directly entailed by their basic mathematical approach. As the cFEP aims to describe cognition within DST, it must describe “meaning” or “belief” as a distribution entailed by a pregiven dynamics and defined over pregiven states of the external world. Because the rFEP relies on information theory to describe the evolution of structure, it must describe “meaning” or “belief” as a property of the relation between mind and world, brought about by the interaction and irreducible to the prior dynamics. In this setting, the description of evolution in cognitive structure is intimately linked to a relational theory of meaning—in line with Varela et al.’s [38] own argument. Taken together, these observations could suggest many different epistemological and ontological interpretations—some of which are warranted, some of which are less so.

In particular, we must clarify what explanatory value we can plausibly assign to the rFEP, given the constitutive role it gives to relationally defined observables. In the context of the natural sciences, explanation is typically understood to proceed from the description of “real structure”—either understood as a formal dependency between observables or as causal interaction between elements of a system [109]. But, the rFEP precisely describes the endogenous construction of novel structure within a system. Thus, neither empirical nor causal relations are held to be invariant within its formalism. To understand exactly what it provides, we need to engage in more detail with the structural realist approach to explanation we discussed in the introduction. As described by da Costa and French [110], this approach relies on the definition of a partial isomorphism between a natural system (the target) and a formal system (its model). This enables a well-behaved structural realist approach to explanation, where our model explains all of the structure its formalism does in fact capture and nothing more [51,52]. Critically, this approach is specifically intended to be agnostic regarding the choice of representation of a given system, given Worrall’s [50] goal of preserving scientific realism in the face of theory change. Thus, the definition of explanation and representation from a *partial* isomorphism between a formal and a natural system is not only an expression of epistemic difficulties in framing a full description of a system. It is also, and most importantly, a positive claim regarding the abstraction inherent in scientific representation.

On this basis, we may consider how this approach applies to the more complex cases of biological and cognitive systems, whose structure itself appears to change in time. To be clear, the claim that several different models may adequately capture the same natural structure does not entail that a given model may adequately capture a change in natural structure. Nonetheless, the fact that the structural realist approach is specifically premised on a partial description of structure gives us ample room to provide a coherent construction for such a model. Indeed, without the assumption that all models of a system should be subsumed by an underlying master model, we may simply work under the premise that some systems indeed evolve in time and attempt to study this evolution. To the extent that we can mathematically capture the structure of that process, we may explain the structure of structure change. (We should note here a semantic creep in the definition of the word “structure” between the various traditions we draw from: while Varela [37] or Rosen [44] define “structure” as the material instantiation of a system, SR defines it as any property of a system that can be described mathematically. Thus, there is no contradiction in terms in studying the structure (in the SR sense) of structure (in the biological organization sense) change or the structure (in the SR sense) of organization itself). Importantly, this approach need not assume that this model should strictly describe causal properties of the target system. Indeed, Saatsi [111] and French and Saatsi [112] have argued that the description of a system’s symmetries constitutes the basis of proportionate physical explanation. But, as we have seen, symmetries themselves constitute a relational concept, capturing structure in the form of a group of transformations which are visible in a representation of the system but not in another. By extension, there is nothing intrinsically suspect in explaining how the thermodynamic irreversibility of observation may lead to the evolution of the constitutive symmetries of a system if we can propose a mathematically coherent way to represent this process.

Thus, the question of whether rFEP constitutes a proper structural explanation of (ontic or apparent) structure change most reduces to the two following questions: is the formal approach proposed by Fields, Friston, et al. [46] warranted? And does it indeed capture the structure underlying some phenomena of interest? On the first ground, the only unusual aspect of the rFEP is its representation of observation as a contextual process, which (as we discussed in the previous section) is grounded in Fields [47] and Fields and Glazebrook’s [48] thermodynamical treatment of the preparation-and-measurement process. Thus, if and where engagement with the world entails an irreversible symmetry breaking associated with system identification, we should indeed expect the emergence of novel observables as constitutive variables of the system and the associated evolution in structure. On the second ground, we should carefully consider when and where contextuality per se appears as a constitutive, explanatorily necessary aspect of evolution in cognitive structure. Not all aspects of cognition that could appear “contextual” in an intuitive sense need to be contextual in the formal sense of the term, nor do they warrant an interpretation in terms of structure change. For example, many of the deviations from rationality in decision theory documented by Tversky and Kahneman [113,114] can be explained by context-dependence in action and perception [115,116] and even modeled meaningfully through a von Neumann algebraic reconstruction of probabilistic thinking [117,118,119]. However, the associated experiments were shown to remain compatible with direct causal influence rather than contextuality per se [120,121].

To clarify this discussion, and where the distinction applies, we propose three distinct definitions of “contextuality”. Contextuality_1_, or context dependence, corresponds to the most intuitive sense of the term in the context of cognitive psychology. It simply describes an influence of context on observable behavior, as documented in numerous experimental paradigms. Contextuality_1_ entails no deviation from a causal realist intuition, and it could, for example, be captured by the integration of perceptual stimuli in a multimodal hierarchical Bayesian architecture with path dependency (as described by the cFEP). Contextuality_2_, or weak contextuality, describes nothing more and nothing less than the generally accepted technical sense of the term: non-commutativity between relevant evolution operators, as captured by the von Neumann algebra formalism. Although it is well known to be incompatible with direct causal influence since Bell [88,89] and Kochen and Specker’s [90] derivation, it is shown to occur in some perceptual decision tasks via standard statistical tests [122,123,124]. Contextuality_3_, or strong contextuality, is the form of contextuality we discussed in relation to Fields, Friston, et al.’s [46] rFEP and Fields et al.’s [97] broader information flow formalism. It does presuppose a breakdown in commutativity of the relevant evolution operators, but, most importantly, it describes the irreducible (re)production of the system’s structure due to the emergence of novel observables as constitutive variables of the system’s activity—that is, a fundamental deviation from the Newtonian paradigm, sensu Rosen [43,44], grounded in a relational definition of the system itself. (Its difference with contextuality_2_ arguably flows from the fact that, unlike Rosen’s [45], Born et al.’s [57] and von Neumann’s [59] formalism treats observation as external to the system they describe rather than being itself a physical process to be explained—as repeatedly noted by Rosen [45] himself).

Although no standard statistical test exists to demonstrate its occurrence, we would carefully suggest that it constitutes (in some form or another) a necessary basis for the explanation of many well-documented phenomena in the science of life and mind. Kauffman [125,126] notes that although Schrödinger [127] explains the heritability of innate traits intrinsic in life, biological existence nonetheless involves the constrained extraction of work towards the maintenance and development of the organism—that is, biological function, which is by construction defined in relation to the system’s organization. Fields and Levin [91,92,108,128] recruit the reference frame framework discussed in Section 4 to explain functionality in biological activity and development by making specific observables “make sense” to the organism as specific targets of regulation. (See also the recent neuromorphic implementations of the broad principle of reference frame reconstruction by Gao, Shi, et al. [129]; Gao, Song, et al. [130], which offers the potential of studying its statistical markers in controlled settings). Building from Levin’s [131,132,133,134] prior work on biological agency and bio-electric computation, Guénin--Carlut [135] argues that contextuality_3_ per se is a necessary explanatory basis for the balance between robustness and adaptiveness in biological development. Relatedly, Guénin--Carlut [136] suggests that contextuality_3_ per se is a necessary explanatory basis for explaining open-ended evolution in human socio-material practices. Although articulated in a different vocabulary, all these arguments flow from the same basis. If one is to claim that a *novel* trait (understood as a trait that was not specified in a pre-existing phase space) emerges, or that it takes a special functional quality *in the context of* an organism, one must specify a mechanism by which that property emerges. Within our typology above, a mechanism which brings about novel states or regulatory qualities verifies by definition contextuality_3_.

At the same time, we must be careful not to overstate what contextuality_3_ is intended to capture specifically. Although it does describe by construction a deviation from the Newtonian regime of explanation as described by Rosen, [43,44], contextuality_3_ does not suggest a breakdown of the generally accepted laws of physics. For example, we may readily observe that humans develop a mastery of specific languages over the course of their life, enabling them to regulate their behavior against otherwise meaningless auditory and visual signals and engage with it in a structured manner. At the same time, we may readily acknowledge and study this phenomenon without presupposing Hameroff and Penrose [137]-style quantum coherence between neuronal states or a commitment to Connes and Rovelli’s [55] thermal time theory as a background cosmology. To recall here again Varela’s [37] distinction, it is simply that linguistic actions make sense to us in the context of our cognitive organization, which is not entailed by the mere description of the underlying physical structure. The same argument applies just as well to the functional states emerging from biological regulation. On this basis, we suggest that the observables brought about as significant variables of biological or cognitive regulation are *real*, in the sense that they provide the basis for real structure that can adequately be studied by the usual means of science, but also *participatory*, in the sense that they are indeed brought about as significant variables by the very process which observes them in the context of biological or cognitive organization. As per Froese [138] and Guénin--Carlut [139], this position is known as *participatory realism*.

Much like the cFEP provides us with a normative principle explaining how a system (as described by its physical structure) may self-organize so as to perform inference within a well-defined perceptive/regulatory space, the rFEP intervenes here as a normative principle explaining how a system (as described by its organization) may produce this perceptive/regulatory space in the first place. As agents observe specific aspects of the world, they necessarily create a symmetry breaking between the aspects of the world that they engage with and those they do not engage with. The resulting entropy exhaust induces the non-separation of reference frames across scales in their own organization and in the broader agent–world dynamics, thus locking in the specific observables they were engaging with at the expense of other possible observables—as per Fields [47]; Fields and Glazebrook [48]. The overall process can be represented as the construction of a communication channel between agent and world as per Fields, Friston, et al. [46], thus providing a mechanism bringing about those observables as classical states of the boundary—as it applies specifically to the organizationally relevant states of the interaction. However, this picture does not tell us much in the absence of an adequate physical language that could bridge the description of organization with that of the underlying physics. In this regard, we will investigate a proposal drawing from Guénin--Carlut’s [19] study of socio-material organization, defined as a system of *constraints* over attention which synchronically determine how an agent engages with the world while being diachronically reproduced by this very process of engagement.

## 6. Cognitive Organization as a Closure of Constraints

We have noted in the introduction the natural synergy between Maturana and Varela’s [140] and Rosen’s [44] respective conceptions of life. Both were highly influential in their approach to biological organization and, together with Kauffman’s [141] seminal study of autocatalytic networks, collectively constitute the central references of virtually all scholarship in this field up to this day. All describe the constitutive property of life as its ability for self-production, and all do so in a way that questions in one form or another the coherence of a Newtonian approach to life (there is room for reasonable disagreement regarding whether this characterizes Kauffman [141] in isolation, but Kauffman’s [125,126] later reinterpretation of his own work in Kauffman [141] constitutes by far the most explicit of the three on this matter)—that is, an approach based on the formulation of well-defined laws of motion in well-defined state-spaces. While we have proposed the rFEP as a relational complement to the cFEP which represents (as far as possible) deviations from the Newtonian regime, its abstract mathematical framing in terms of reference frames and observables may raise doubts concerning its applicability to concrete issues—if only by making its central intuitions quite inaccessible to most experts in cognitive and social sciences. Thus, we will dedicate this section to articulating the positive insights which emerged from the biological organization literature and hopefully provide a more accessible articulation of how we should understand deviations from the Newtonian regime in biology and cognition. To do so, we will first articulate what is to our knowledge the very first articulation of a recognizably relational view of life: Varela’s [37] generalization of Maturana and Varela’s [140] autopoiesis as *organizational closure*. (We should note that Maturana and Varela [140] constitutes the canonical citation for a collection of two essays initially published in 1970 and 1973, respectively. Thus, Varela [37] is indeed a later articulation in terms of intellectual trajectory).

Varela’s [37] central goal is to investigate the properties of biological autonomy, understood to constitute the ability of biological systems to self-produce and self-regulate. Its main contribution is to reframe Maturana and Varela’s [140] account of autopoiesis around the distinction introduced in Varela ([37], Chapter 2) between the abstract relations defining the system’s *organization* and the material *structure* instantiating these relations. On this basis, Varela ([37], Chapter 7) defines *organizational closure* (the term *operational closure* is generally used as a synonym in the later literature) as the property of systems defined by a network of mutually dependent processes collectively constituting it as a recognizable unit (p. 66). Varela ([37], Chapter 7) further formulates the *Closure Thesis*, that is, the conjecture that all autonomous systems must verify organizational closure, and suggests that they should be understood through the study of this property. Varela ([37], Chapters 12, 13) then formalizes organizational closure as the action of recursive processes, represented under the form of algebraic operators. Such processes naturally define the system’s organization as their fixed point and, in addition, define its cognition as the structural couplings with the world it enters through those processes ([37], Chapters 7, 16). Overall, this perspective conceptualizes self-production and behavioral control as two dual perspectives on the same fundamental processes, driven by the central (and mechanistically enforced) imperative of maintaining organizational closure ([37], Chapter 10). Autopoiesis then emerges as the special case where operational closure concerns biochemically implemented and spatially bounded processes, in which case Maturana and Varela’s [140] intuition about the relation between biology and cognition re-emerges in full.

Intuitively, Varela’s [37] organizational closure may appear as a quasi-synonym of Rosen’s [44] closure to efficient causation. After all, both describe biological organization as a closure relation by which a system is producing itself as what it is. Nonetheless, this would be missing important nuances between the two approaches. Rosen [44] indeed considers formal entailment relations between components of a system, while Varela [37] is interested in causal relations of production between processes within a physical structure. Most importantly, Rosen [44] argues explicitly for breaking with a mechanistic approach to account for teleology in describing biological organization, while Varela [37] precisely attempts to reconstruct normativity from a mechanistic approach excluding any reference to teleology. Nonetheless, the later analysis of these approaches to biological organization proposed by Mossio and Moreno [142] or Montévil and Mossio [54] identifies the same limitation with both. Indeed, both Varela [37] and Rosen [44] ultimately frame life in terms of abstract algebraic structures and thus do not offer a clear distinction between the self-production of life and that of simpler autocatalytic structures such as the flame of a candle or the hydrological cycle. Ironically, this opens both to the same criticism of non-specificity extended towards the FEP by Bruineberg, Kiverstein, et al. [10] or Nave [41,42]. Rather, Mossio and Moreno [142] and Montévil and Mossio [54] suggest explicitly grounding the study of biological organization in the thermodynamics of the work cycle which concretely maintains and (re)produces biological structure, as documented by Kauffman [125,141]—while remaining vigilant not to identify biological organization with the mere description of the flows themselves.

On this basis, Montévil and Mossio [54] and Moreno and Mossio [53] have proposed an alternative conceptualization of biological autonomy centering the description of how biological systems may extract the necessary work from ambient thermodynamical gradients to maintain and (re)produce themselves as a whole ([53], Section 1.2). More precisely, Moreno and Mossio ([53], Section 1.3) define a constraint as a system which induces a symmetry breaking in the form of some thermodynamical process while remaining invariant at the characteristic time-scale of the process. This simple time-scale separation between constraints and processes enables a dual description of constraints as *causes* defining the dynamics of a process at a given time-scale and *products* of other processes at a longer timescale. For example, the vascular system is at the same time the (efficient) cause for the form of blood diffusion at the scale of seconds and the product of the vascular tissues’ self-production at the scale of days and months. In turn, the mutual relation of (re)production between many constraints distinguishes the self-production of living systems from other processes governed by the self-organized dissipation of a single, externally imposed thermodynamic flow ([53], Section 1.4). Thus, biological organization can be defined as the *closure of constraints*, the property of systems of constraints where all constraints are produced by and producers of at least one other constraint in the system ([53], Section 1.6). This approach reproduces the core insights from Varela [37] without assuming a priori the abstraction of biological organization into a well-behaved algebraic or dynamical systems-theoretic structure (Table 1).

Moreno and Mossio ([53], Chapters 2, 3) further articulate how this regime of organization grounds a relational account of biological emergence and teleology, defined as the action of the mutual causal interaction between constraints without assuming the existence and causal efficacy of the organism as a whole. Moreno and Mossio ([53], Chapter 4) discuss in more detail the interactive dimension of agency, as autonomous systems act in the world along goals and norms generated by closure. However, Moreno and Mossio ([53], Chapter 7) deviate from Varela’s [37] strong life–mind continuity by describing the evolution of neural systems as the development of a new level of autonomous organization, dynamically decoupled from although causally intermeshed with metabolism. In their view, such specialized, autonomous regulation subsystems enable more complex forms of adaptive agency, such as the integration of neural constraints through development and social interaction (Section 7.6). Given Moreno and Mossio’s [53] non-mathematical approach, they do not prescribe a specific manner to represent the development of such neural constraints or their effect on cognition. However, their definition of constraints as physical systems which causally act within a system so as to implement a symmetry breaking in its process invites a fairly straightforward analogy to the mathematical framework of gauge theory. Specifically, we can understand the action of a system of constraints on a set of processes as a gauge force corresponding to the symmetry group it entails, where the gauge symmetries are produced within the system rather than assumed a priori [19].

Given the considerations we developed in the previous sections, the symmetry-theoretical framing of Moreno and Mossio’s [53] closure of constraints framework entails a quite direct synergy with Fields, Friston, et al.’s [46] rFEP. Indeed, the rFEP describes contextual observation as a process of thermodynamically grounded symmetry breaking, which is understood to bring about novel observables as causal variables of a system’s interface with the world. This provides a natural account of the physics underlying the production of constraints (i.e., materially instantiated symmetries) within Moreno & Mossio [53]’s framework. In particular, assuming that it does ground the emergence of contextuality_3_ from the thermodynamics of observation in biological systems, it would provide an adequate physical ground to describe and explain the open-ended nature of biological evolution and development [135,136]. Conversely, Moreno and Mossio’s [53] closure of constraints framework provides a natural definition for the reference frames through which Fields, Friston, et al. [46] describe a system’s engagement with the world. Indeed, we can define the reference frame of a given observation as the collection of each organizationally relevant outcome it may have in the context of the organization—thus defining symmetries sensu Rosen [45] relating the equivalence of micro-states having the same outcome. In this context, the closure of constraints can be given a dual, informational characterization: each constraint of the organization must be part of the reference frame defined by the broader organization so that its presence (or absence) can be observed and brought about by the action of the system; and it must be necessary to define the reference frame that observes at least one other constraint within the system.

To be clear, Moreno and Mossio [53] would perhaps object to the explicitly information-theoretic framing adopted by Fields, Friston, et al. [46] and the associated account of biological organization [92,108,135]. Indeed, Moreno and Mossio ([53], Section 1.2) cite a warranted skepticism of the conceptual dualism between material and informational description as a core motivation for their overall framework—a skepticism echoing the earlier commitments of Varela [37], Maturana and Varela [140], and Varela et al.’s [38] approach to agency. Nonetheless, we need to emphasize that the kind of informational approach which structured early computational functionalism is quite different from that which we propose here. And, incidentally, the form of computational functionalism criticized by Varela et al. [38] was characterized by the assumption that neuronal states should be equipped with intrinsic semantic value given the broader organization so that neuronal dynamics would naturally correspond to logical operations in a predefined algebra. On the contrary, the rFEP (and the broader physics of biological computation we discussed in Section 3) proceeds from the bare thermodynamics of interaction between two systems to derive what states may be equipped with semantic value given their prior organization and how this may in turn affect the evolution of their organization and of the physical structure it recruits. In other words, the rFEP provides a thermodynamically grounded solution to the problem of groundlessness raised by Varela et al. [38] and does so by capturing the very participatory, relational nature of sense-making they argued was disregarded by early forms of computational functionalism.

In this approach, the concept of constraints affords a reframing of the cFEP/rFEP framework we discussed in Section 4 in terms of actual, observable physical objects whose existence, causal efficacy in synchronic dynamics, and diachronic constitution are manifest in the world we can observe. Indeed, a well-defined system of constraints acting over a well-defined set of processes so as to entail a well-defined symmetry group over its dynamics can unambiguously be described in Newtonian terms. Thus, the cFEP can faithfully capture the synchronic emergence of behavioral regulation given the system of constraints characterizing a biological organization at a given point in time. Conversely, the symmetry breaking entailed by the choice of reference frames (both at nested scales within the organization and in its engagement with the world) described by the rFEP can be understood as the self-constitution and (where novel constraints emerge as constitutive elements of the closed system) the diachronic evolution of such systems of constraints. Importantly, this framing dissolves the tension between the supposedly Newtonian domain of physics and a proposed account of diachronic change violating this Newtonian premise, which was manifest in our discussion in Section 4. Indeed, Moreno and Mossio [53] at no point assume that a closed system of constraints and its action on biological processes should have a natural mathematical representation. The statement of our cFEP/rFEP framework applied to biological constraints then reduces to the following, independently unproblematic and mutually coherent premises: (1) biological organization can be described in terms of constraints over biological processes; (2) the synchronic action of those constraints can be described in Newtonian terms; (3) the diachronic evolution of those constraints can be described in terms of what novel facts are brought about as significant to their collective self-action.

## 7. Conclusions

In this article, we have explored the possibility of a truly relational formalization of life and cognition and its relation to the known physics of cognition. In Section 2, we have discussed Rosen’s [43,44,45] foundational work on the limitation of mathematical representation for natural systems and, more specifically, on the role of observation and interaction in defining the dynamics of any given system. In Section 3, we have applied this insight through a cursory look at the thermodynamics of observation in biological systems, leading up to Fields’s [47] and Fields and Glazebrook’s [48] derivation of contextuality from the very symmetry breaking intrinsic to observation. In Section 4, we have discussed in depth the formalization of this process proposed by Fields, Friston, et al. [46] and argued it constitutes a *relational* complement to the classical formulation of the FEP by Friston [6]. In Section 5, we have addressed the most salient critiques against the associated ontic regime of *participatory realism* and suggested that it simply constitutes an application of structural realism to systems verifying *contextuality_3_*—regardless on the ultimate ontic stance one may adopt on the “reality” of the observables. Finally, in Section 6, we have reframed these insights around Montévil and Mossio’s [54] and Moreno and Mossio’s [53] account of biological organization as a closure of constraints, suggesting that the cFEP captures the dynamics of cognitive engagement entailed by a well-defined system of constraints while the rFEP captures the evolution of a system of constraints through the emergence of novel observables through its own action.

For the sake of clarity, we now need to assess the nature of those arguments against the classical formulation of the FEP. Let us emphasize that none of the arguments presented here should be understood as an invalidation or even a criticism of Friston’s [6] formalism. Rather, it simply constitutes a discussion of the limitations entailed by its mathematical framing within Dynamical Systems Theory. By construction, DST captures with maximal generality the evolution of well-defined objects in well-defined spaces along well-defined dynamics—nothing more and nothing less. Much attention has been given to the question of whether or not biological systems *really* are defined by the mathematical structure of DST. But, as we have discussed in Section 2, such a question is ultimately undecidable from an empirical perspective given Rosen’s [45] results on the observation and composition of general dynamics and also irrelevant to the question of whether it can provide a mathematical explanation for any given aspect of cognition. By our account, it seems to capture its *synchronic* dimension—what organization of meaning is enacted by a system equipped with a given structure—but not its *diachronic* dimension—how this structure changes through engagement with the world. Our main contribution in the present discussion is to suggest that both the question of biological realism and the synchrony–diachrony integration can be satisfyingly resolved simply by reframing the FEP in terms of observable physical entities rather than a supposedly intrinsic mathematical structure: namely, in terms of *biological constraints* as defined by Montévil and Mossio [54] and Moreno and Mossio [53].

Indeed, and unlike a priori mathematical structure, physical entities such as constraints are not part of a Platonic world of ideas free from change, ambiguity, and the diachronicity intrinsic in development. In particular, we can very plainly observe change in biological constraints through the processes of development, aging, learning, regeneration, etc. This requires a slightly different understanding of the explanatory value of the FEP and, most importantly, enables an explicit consideration of the complementarity between Friston’s [6] cFEP and Fields, Friston, et al.’s [46] rFEP. The dynamical systems-theoretic definition of the cFEP can be understood as the causal structure of a given set of processes (corresponding to the state variables of the model) given the underlying system of constraints (defining the symmetries of the system and thus the space of relevant variables and the dynamics they verify). But, this model in itself is not an a priori or sufficient representation of the system, as the constraints are themselves produced (and potentially transformed) by the very same process they give shape to. To capture this latter dimension, we propose to recruit the rFEP, whose information-theoretic definition captures the constitutive symmetry breaking which defines the organization of the system, and thus the evolution of its organizationally relevant structure. Indeed, the selective openness of the biological organization can be understood as observation/engagement with itself and the environment through reference frames, defining what states of the world are brought about as “observables” (constitutive, irreducible variables in the system’s organization) in virtue of the symmetry breaking entailed by this selective openness.

To be clear, we do not claim that the present article articulates a fully coherent mathematical framework apt to extend or replace Friston’s [6] cFEP. To do so would require a deep dive into the complementarity of dynamical and relational definitions of the same system, framing “observation” as a meta-dynamical operation taking a system from one DST representation to another. The natural mathematical framework to do so would be category theory, taking the DST representation as a category defined by the action of the flow over subspaces of the state-space and the relational process of observation as the functor between those categories—more likely than not encoding a non-commutative notion of time *à la* Rosen [43], with the complexities this entails. (We should note here the distinction between the rFEP itself, which is framed in terms of non-commutative observations in commutative time, and the hypothetical integration of the cFEP and rFEP within a single mathematical framework. In the latter case, the non-commutative process of observation operationalizes evolution in the space of possible structures and thus encodes a natural notion of time as it is experienced by the system). But, doing so rigorously would require a much greater degree of formalization than we can afford here given our present objective of accessibility and conceptual clarity. Thus, we should simply note that an active inference-informed, category theoretical approach to cognition is not without precedent. Smithe’s [143,144,145] Bayesian lens theory and Albarracin et al.’s [146,147] subsequent formalization of shared protentions under active inference constitute a straightforward instance of this—although the underlying physical intuition is much less straightforward than Fields, Friston, et al.’s [46] rFEP. Rather than commenting and building on these approaches, our contribution here begins and ends with a comparatively modest (and mathematically light) analysis of the structural properties of the cFEP and the rFEP which enable them to play complementary roles in grounding our understanding of biological and cognitive organization.

While more open to debate on philosophical grounds, this approach enables us to reframe our argument into a few conceptually accessible insights. First, the organization of cognition can be represented as a system of *constraints* embedded across an agent’s internal architecture and its socio-material context. Such constraints collectively produce the flow of cognitive engagement with the world, thus underlying its synchronic representation in terms of Dynamical Systems Theory. However, no assumption should be made regarding the sufficiency of such representations, as the system of constraints characterizing an agent is bound to evolve through its own activity (notably, through learning, aging, regeneration, etc.). Second, the diachronic evolution of cognitive organization can be captured by the property of *contextuality*—or, more precisely, contextuality_3_ as we defined it in Section 5. This property can in turn be described at two distinct levels, which capture two dual aspects of biological computation as we have described it here. At the phenomenological level, contextuality describes the capacity of biological organization to “observe” novel states of the world by regulating its activity against them, thus bringing them about as constitutive variables of its dynamics. At the organizational level, it describes the corresponding reproduction of the system of constraints producing (or perhaps being produced by) those novel observations. This dual intuition can be applied at all scales of biological and cognitive behavior if we accept the relational aspect of the underlying organization—and in doing so, it seems that we would dissolve much of the tension between synchronic and diachronic accounts of mind and life.

However, the most notable aspect of our argument is perhaps its defense of a purely physicalist and classical understanding of the rFEP-associated research program describing biological activity through contextual observation under specific reference frames [92,93,108]. Indeed, the property of contextuality is generally understood as a mark of the quantum realm and thus incompatible with the known classical physics of life and mind. However, as we noted in Section 3, Fields [47]; Fields and Glazebrook [48] proposed a purely classical mechanism for the emergence of contextual observation in the context of repeated measurement. And, as we noted in Section 6, the reference frames this model presupposes can be provided by the relation between constraints entailed by Moreno and Mossio’s [53] theory of biological organization as a closed system of constraints. The critical observation underlying that argument is that, to borrow here again Varela’s [37] structure–organization distinction, the “contextuality” of biological systems does not concern their *structure*—the basic physical processes in which they are implemented. It concerns strictly their *organization*, i.e., the system of physical relations within that structure which bring about specific facts as relevant to the integrated activity of the system. Thus, our framework does not presuppose that the physics underlying biological processes must break with causal realism or otherwise violate classical intuition. It merely describes the biological organization as a relational system which indeed may bring about some aspects of the world as relevant to its activity while grounding this description in the bare thermodynamics of observation in biological systems.

## Figures and Tables

**Table 1:** A comparative table of the main theories of biological autonomy.

	Varela [37]	Rosen [44]	Kauffman [125,141]	Moreno and Mossio [53]
Target	Biological self-production	Biological self-production	Biological self-production	Biological self-production
Core concept	Organizational closure	Closure to efficient causation	Autocatalytic sets + work cycle	Closure of constraints
Definition	Abstract/algebraic relations between physical processes	Abstract/algebraic relations between physical processes	Physical processes	Physical relations between processes/symmetries
Stance on mechanicism	Explicit mechanicism	Explicit anti-mechanicism	De facto mechanistic	De facto mechanistic
Newtonian approach	Implicit rejection	Explicit rejection	Explicit rejection	Implicit rejection

## Data Availability

No new data were created or analyzed in this study. Data sharing is not applicable to this article.
